# Do high aspirations lead to better outcomes? Evidence from a longitudinal survey of adolescents in Peru

**DOI:** 10.1007/s00148-021-00881-y

**Published:** 2022-01-28

**Authors:** Carol Graham, Julia R. Pozuelo

**Affiliations:** 1 The Brookings Institution, Washington, D.C. USA; 2 University of Maryland, College Park, MD USA; 3 Gallup, USA; 4 Department of Psychiatry, University of Oxford, Oxford, UK; 5 Centre for the Study of African Economies, University of Oxford, Oxford, UK; 6 MRC/Wits Rural Public Health and Health Transitions Research Unit (Agincourt), School of Public Health, Faculty of Health Sciences, University of Witwatersrand, Johannesburg, South Africa

**Keywords:** Adolescents, Aspirations, Human capital outcomes, Risky behaviors, Peru, D91, I12, I25, I3

## Abstract

Using a novel panel survey of relatively poor urban Peruvian adolescents, we explore the link between educational aspirations and propensity to invest in the future. Aspirations comprise hope and agency. We find remarkably high educational aspirations, even among relatively poor individuals and adolescents who were exposed to negative shocks, suggesting high levels of resilience. We also find high occupational aspirations and aspirations to migrate. High-aspiration respondents were also more likely to invest in their education and avoid risky behaviors. These are associations as we do not have enough data to establish causality, although we were able to control for within-person traits. Aspirations are stable over time and positively associated with personality traits such as self-efficacy and life satisfaction, which help explain their persistence over time. Our findings complement those of other recent studies that highlight the role of personality traits in addition to cognitive skills in long-term educational, health, and socioeconomic outcomes.

## Introduction

Adolescence is a period of exploration in which individuals start to develop their self-identity and make important decisions about their future, ranging from education, relationships, and entrance into the labor market to health behaviors (Sawyer et al. 2012). Risky behaviors such as unsafe sex, binge drinking, and delinquency tend to emerge during this period, potentially jeopardizing those plans (Pozuelo et al. 2021; Steinberg 2004). Poor self-concepts (such as esteem) and hopelessness are also significant risk factors for adverse health behaviors during adolescence and adulthood (Mann 2004). Although aspirations are at the heart of many behaviors as well as a subject of interest in behavioral sciences, we know little about how aspirations shape those behaviors and subsequent accomplishments. Better understanding this relationship is particularly important for adolescents, who are at a point in their lives when aspirations will likely guide their choices about the future.

Several studies in the literature on the economics of well-being have found that people with high hopes and aspirations tend to have better outcomes in a range of areas from health to the labor market to social arenas (Graham et al. 2004; De Neve and Oswald 2012; O’Connor and Graham 2019). Longitudinal studies with adolescents find that aspirations predict future educational and occupational attainment, as well as engagement in risky behaviors (Beal and Crockett 2010; Mahler et al. 2017; Schmid et al. 2011; Sipsma et al. 2013).

In contrast, lack of hope for the future—often due to the daily stress that accompanies poverty and makes future planning difficult—can contribute to inferior later life outcomes as individuals who do not believe in the future have higher discount rates and are less likely to invest in their futures, such as via education and savings (Mullainathan and Shafir 2013).

It is also possible that high aspirations can result in frustration if the aspired goals are unattainable. A study based on the Young Lives panel study for India finds an inverse *U*-curve in the relationship between aspirations (of parents and adolescents) and education outcomes, with both low and overly high aspirations leading to worse outcomes than those in the “bell” of the curve (Ross 2019). Consistent with this result, another study finds that aspirations that are ahead, but not too far ahead (attainable in a shorter time frame), provide the best incentives for key investments (Ray 2006). The availability of opportunities and the social and circumstantial factors shaping aspirations, meanwhile, can hinder the aspirations of disadvantaged groups (Fruttero et al. 2021).

Using a novel panel survey of relatively poor urban Peruvian adolescents, we explore the link between aspirations and individuals’ propensity to invest in the future. Our analysis has four objectives. First, we aim to explore educational aspirations (and how they compare to occupational and migratory ones) among participants and to understand how aspirations vary with individual characteristics, childhood experiences, and the characteristics of the household in which they grew up. Second, the longitudinal nature of the study allows us to examine how aspirations change over time *within* individuals. When possible, we determine whether adolescents met their aspirations or whether they mis-predicted their futures. Third, we look at the link between aspirations and broad personality traits such as self-efficacy, subjective well-being, and locus of control. Lastly, we explore whether high aspirations are correlated with better human capital outcomes. We investigate this by looking at an individual’s propensity to invest in their own future, as measured by education outcomes, time use, and engagement in risky behaviors such as substance use and delinquency.

We designed our survey and the specific measures therein based on our interest in exploring hope—an understudied but important trait—combined with agency as core features of aspirations and the outcomes they lead to. Our study is distinct from others in that we explicitly collect data on the focused aspirations and the personality traits of our respondents at two points in time, with the objective of understanding the interactions between them. While we see aspirations as focused on specific goals, we also see aspirations as integrally linked with hope and its implicit link to the pursuit of a better life in the future. As such, our survey includes several questions that reflect the domains of the “big five” personality traits, as well as other questions that are tailored for adolescents at a point in their lives that they are making critical decisions about their futures. To our knowledge, this is one of the few surveys of its kind, with the benefits and risks that come with exploratory data.

This paper also focuses on an understudied and significantly disadvantaged population group (Blum and Boyden 2021). Most of the evidence exploring the association between aspiration and human capital outcomes among adolescents comes from studies conducted in high-income countries (Beal and Crockett 2010; Mahler et al. 2017; Schmid et al. 2011; Sipsma et al. 2013). Evidence from low- and middle-income countries, where 90% of the world’s adolescents live and where there are far fewer resources and support systems available, remains scarce. This study contributes to closing this gap by exploring the association between aspirations and future outcomes among relatively poor adolescents in a middle-income country.

Our results show that aspirations can be very high even among relatively poor individuals and are also resilient to a range of negative shocks in an already challenging context. Indeed, we find that aspirations are quite stable over time, which is particularly notable given that our respondents are in a period in which young lives change and develop a great deal. Aspirations in our sample are positively correlated with personality traits such as self-efficacy and subjective well-being, which help explain their persistence. Finally, we find that high aspirations are strongly correlated with positive human capital outcomes such as higher investments in education and risk avoidance.

## Aspirations and its determinants

Aspirations are commonly defined as a hope or ambition of achieving something. The concept of aspirations spans multiple—often interrelated—dimensions both at the individual level (for example, level of education that one aspires to, type of job, fertility, status) and at the collective level. The concept is different from expectations, which typically encompass an individual’s beliefs about what they think they can achieve with effort (the most likely or realistic outcome) (Dalton et al. 2016). It is also different from hope alone, which, in our view, reflects optimism about the future without explicitly involving agency or clear goals. While aspirations are aimed at specific goals—such as higher levels of education—these can also be a means to achieve higher and less well-defined goals, such as having a better life.

More formally, aspirations have three distinctive aspects. They are future-oriented, as they involve goals to be accomplished in the future. They act as motivators and drivers of effort, as they allow us to narrow our effort and attention toward accomplishing our goals, and away from less relevant activities. Finally, they require some amount of effort to achieve (Bernard and Taffesse 2012).

Aspirations evolve over time, and are shaped by individual characteristics and their experiences, their families, and interaction with the social environment. Aspirations may also interact with objective factors such as capability and talent, leading to virtuous—or vicious—circles. As a result, several factors have been identified as potential determinants of aspirations, as well as possible interactions among these factors.

One perspective, often taken by behavioral economists, is that aspirations are drawn from individual’s past experiences, and, at the same time, are profoundly affected by one’s social environment (Genicot and Ray 2017). According to this view, individuals adjust their aspirations to what is perceived to be possible.

This view has implications for people living in poverty as the lack of opportunity and/or information about what is possible can result in a reduced ‘capacity to aspire,’ frustration if the aspired goals are unattainable, and vicious cycles of continually lowering aspirations in ways which can perpetuate poverty (Appadurai 2004; Dalton et al. 2016). This adaptation may be explained in part as a psychological preservation mechanism for those with limited capabilities or who live in conditions that do not allow them to aspire, as in the case of women in situations with extremely unequal gender rights (Frederick and Loewenstein 1999; Graham 2011, 2009; Ray 2016).

Poor people in difficult conditions may often report to be very “happy”—in the sense of momentary contentment—but they typically score lower on evaluative questions which prime them to think about their lives as whole (Graham, 2009). A more recent literature, meanwhile, focuses on optimism (as opposed to focused aspirations) and finds that some cohorts who lived in deprived conditions are much more optimist than their counterparts of the same income levels. The optimistic ones tend to do better over time in the education and health arenas. This is the case, for example, for low-income African Americans and Hispanics compared to low-income whites in the USA (Graham and Pinto 2019; Kerpelman et al. 2008).

A second perspective is offered by personality and social psychologists (and some economists) who believe that aspirations are linked to but distinct from broad personality traits, which include traits like self-esteem, locus of control, and self-efficacy (Almlund et al. 2011; Bandura et al. 2001; Dercon and Singh 2013). Studies have shown that compared to measures of fluid intelligence such as IQ, personality traits are more likely to evolve over time and to interact with the environment well into the middle ages (Almlund et al. 2011). These personality traits, in turn, are known to predict future outcomes such as education attainment and health and labor outcomes as strongly as do measures of cognitive ability (Borghans et al. 2008; Heckman and Kautz 2012).

Our definition of aspirations fits into the broader concept of traits used by Heckman and Kautz (2012). They use the term “personality traits” to describe the attributes that are not captured by measures of abstract reasoning power, such as IQ. These attributes have many names, including soft skills, personality traits, noncognitive skills, character, and socioemotional skills. These different names connote different properties. The term “traits” suggests a sense of permanence and possibly also of heritability, while the terms “skills” and “character” suggest that they can be learned. Their empirical work suggests that both cognitive and personality traits can change and be changed over the life cycle but through different mechanisms and at different ages.

Heckman and Kautz note, though, that most studies of the role of personality traits in determining outcomes, by both psychologists and economists, neglect to include the role of a deeper set of preferences or goals, which can also be thought of as traits. Achieving such goals requires certain traits, such as intelligence or conscientiousness. Under this view, traits are developed through practice, investment, and habituation, which are in turn influenced by incentives. The apparent stability of expressed traits across situations may be a consequence of the stability of the goals and incentives themselves. Studies that account for the endogeneity of investments as in education provide further evidence of the causal effect of education and cognitive and personality traits on outcomes. As such, human capital outcomes are at least in part endogenous to personality traits.

Heckman and Kautz rely on the so-called “big five” personality traits in their empirical work. These are conscientiousness, agreeableness, openness to experience, extroversion, and neuroticism/emotional stability. While these are largely stable over the life course, they can be influenced by experiences, parenting, and the social environment. . The traits that we use in our survey—such as optimism, self-esteem, belief in hard work, mental states, impatience, and ability to make friendships—have many elements of these five traits. We chose our specific measures based on our interest in exploring the role of hope combined with agency as core features of aspirations. Our selection of personality trait measures was also influenced by whether they had been already tested in psychological studies of adolescents. As such we built from the pre-existing research on personality traits and outcomes but also adapted our metrics to our key questions of inquiry and to the population under study. Given that we focus on adolescents, relations with parents and/or peers may be critical in forming preferences and incentives.

## Methods

### Study context

We collaborated with the Instituto de Investigacion Nutricional (IIN) in Lima, directed by Dr. Mary Penny, to conduct a new panel survey of 400 adolescents in the district of San Juan de Lurigancho. The first study wave was conducted between May–June 2017, when the adolescents were aged 18 and 19 years old, and was followed by another subsequent wave of data in February 2020, with the interviews being completed just before the COVID-19 pandemic hit Peru. Institutional Review Board approval for this study was obtained before each round of data collection from the IIN.

San Juan de Lurigancho is a large peri-urban and relatively poor neighborhood of Lima with a population of over 1 million residing in a 50-mi^2^ area. The district is home to several slums, and to high levels of crime and youth unemployment (Andrade-Chaico and Andrade-Arenas 2019). The adolescents from our survey come from poor or near-poor families. Living standards range from concrete houses with newly acquired piped water and sewage and electricity, as well as access to metro and bus transport, to significantly more impoverished prefabricated homes further away from the center still in the process of acquiring these amenities.

We focused on late adolescence (18–19 years at wave 1) as they are at a point in their lives where they have enough education and experience to observe, and at the same time are at a critical juncture in making vital choices. Most of them (83%) had completed secondary education in wave 1 and were making decisions about their continued education, entrance into the labor market, family formation, and risky behaviors such as sexual relationships and substance use.

In the past decades, the Peruvian educational system has undergone significant transformation, leading to substantive progress in providing access to education,^1^ improved teacher-training programs, and increased education spending. While there has been progress, challenges such as significant differences in access and quality of education across rural and urban areas remain, which in turn show up in the performance statistics (OECD 2016).

As in many other places around the world, returns to different levels of education are changing. In Peru, between 1980 and 2004, returns to primary, secondary, and technical education fell relative to returns to tertiary education. While returns to secondary education halved (from 12.6 to 6.3%) in that period, returns to tertiary doubled, reaching 17.3% by 2004 (Yamada 2006). The aspirations that our respondents have for college and post-college education suggest that they are aware of these differential returns.

While the parents of our San Juan de Lurigancho respondents do not have college educations and are in low skill jobs—such as construction, taxi drivers, domestic servants, and local market owners—the aspirations of their children suggest a strong awareness of the need to get tertiary education to do better than their parents. Informal interviews in the area, meanwhile, suggest that parents play a strong role in this by encouraging them to seek higher education.^2^

### Measures

#### Aspirations

We asked about aspirations in three domains: education (our primary variable of interest), occupation, and migration. Respondents were asked directly about their aspirations in both waves. This approach has been shown to elicit more reliable measurements of individuals’ aspirations when compared to indirect approaches that use other measures such as the self-reported minimum income need to infer measures of aspirations (Bernard and Taffesse 2014).

##### Educational aspirations

We asked participants what level of education they would like to complete. The variable is coded on a four-point scale where 0 corresponds to low aspirations and 3 represent very high aspirations (postgraduate education).

##### Occupational aspirations

We asked participants about the type of job or occupation they would like to achieve in their life. We used ILO’s International Standard Classification of Occupations (ISCO-08) to rank our respondent’s occupational aspirations. Scores range from 0 (elementary occupations) to 8 (managerial occupations) (ILO 2012).

##### Aspirations to migrate

We also asked adolescents whether they would like to migrate somewhere, and if they did, we asked where. The variable is coded on an 8-point scale, where the score indicates how far a respondent aspires to migrate (0 = no desire to migrate, 7 = aspirates to migrate somewhere abroad).

#### Personality traits

##### Emotional symptoms

These are measured using the 5-item subscale of the strengths and difficulties questionnaire (SDQ), one of the most widely used screening instruments to measure internalizing problems among young people (Goodman 1997). The scale assesses symptoms such as headaches and stomachaches, worry, unhappy/tearful, nervous, and fears.

##### Locus of control

We selected 4 items on locus of control from Levenson’s (1974) original scale (Levenson 1974). Two of these items measured internal locus of control, while the others measured external (powerful others and chance) locus of control. All the above items were measured on a Likert scale, from strongly disagree (= 0) to strongly agree (= 3). The scale evaluates which forces individuals consider as determining their lives.

##### Self-efficacy

We used 5 items from Schwarzer and Jerusalem’s (1995) Generalized Self-Efficacy Scale. For each item, participants could choose from ‘not at all true’ (score = 0) to ‘exactly true’ (score = 3). The scores for each of the five items were summed to give a total score. This construct measures an individual’s general confidence to cope with unforeseen or demanding situations (Schwarzer and Jerusalem 1995).

##### Life satisfaction

This measure, which assess current satisfaction with life, is based on responses to the best possible life Cantril ladder question. This commonly used question asks respondents to place themselves on a 9-step ladder in which they compare their lives to the best possible life they can imagine.

We also measured respondents’ beliefs that hard work will get them ahead, participant’s willingness to take risks, sociability, self-esteem, and optimism. We measured impatience by administering the classic discount rate question, where respondents decide between immediate sums of money today versus higher sums in the future.

Both our measures of locus of control and of impatience—and their strong association with our definition of aspirations—have a number of similarities with Hofstede’s (2001) concept of long-term orientation, which he saw as varying across both people and cultures. In contrast to Hofstede, though, we focus on differences in these traits within a homogenous population rather than differences across cultures. The works of Galor and Özak (2016) and Figlio et al. (2019), which focus on long-term orientation and outcomes in terms of risky behaviors (smoking), saving, and education outcomes are more directly related to our analysis and our measures of time preference and ability to delay gratification, which in turn are part and parcel of focused aspirations.

#### Individual and household characteristics

##### Socioeconomic status

We measured respondent’s socioeconomic status using a household asset index which included several questions on housing quality, access to services, and ownership of consumer durables. We created a weighted average, where a higher wealth index indicates a higher socioeconomic status.

##### Negative shocks

We collected data on six different types of negative shocks: robbery, whether the participant suffered from an accident (defined as serious injuries that would prevent respondents from doing their normal activities and/or require medical attention), sickness of oneself or a family member, death of family member, whether one (or both) parent left the household, and unemployment shock or natural disaster.

We also included a battery of questions on education, health, family support, and employment.

#### Human capital outcomes

##### School outcomes

These were assessed by the highest level of education attained by our respondents and whether they are full-time students.

##### Time use

We asked respondents how much time they allocated to school-related tasks and whether they participated in any professional development training, such as language courses.

##### Risky behaviors

In a separate and self-administered section, we asked respondents about their sense of self-respect in their interactions with parents and peers; their usage of cigarettes and alcohol; and their attitudes about risky sexual behaviors and delinquency, and their proclivity to those, among others. Answering these questions was optional, and respondents provided their answers to the interviewers in a sealed envelope.

### Statistical analysis

To explore whether high aspirations result in better future outcomes, we take a two-step approach.

In Model 1, we use a lagged, dynamic model to explore whether aspirations at wave 1 are correlated with future outcomes at wave 2. We control for a range of individual- and household-level characteristics, as well as personality traits.

In model 2, we exploit the fact that we have a panel study to estimate an individual fixed effect model. The fixed effect model eliminates one major source of confounding by controlling for any unobserved time-invariant variables that may be correlated with the explanatory variables (Wooldridge 2010). That is, in a fixed effect specification, constant variables (observed or unobserved) such as sex, parental education, and ethnicity are dropped out of the model, thus eliminating any concerns we might have about their potential confounding effects. Although this model is not sufficient to claim any causal relation, it allows us to get a better identification of the relationship between aspirations and human capital outcomes, while controlling for within-person traits.

While model 2 may be more precise, model 1 allows us to explore the dynamics underlying the later-term outcomes of our adolescents. More information about each model, along with the model specifications, can be found in the appendix.

## Results

### Basic socio-demographics and attrition analysis

Table 1 shows the main descriptive statistics across both waves of data. Mean average age is 18 years old at wave 1, with the sample being evenly split between males and females. Levels of education are relatively high, with only 3% of the sample having no formal education in wave 1. By wave 2, more respondents had married, had children, and lost a parent. Average years of education increased, but so did the number of adolescents that dropped out of school. Overall, half of the sample was no longer enrolled full-time in education at wave 2. Respondents reported that the main reason for leaving school was lack of financial resources (for example, respondents could not afford school and had to look for a job to make a living). Most respondents experienced some form of negative shock (90.5%). The most common shocks were thievery, followed by parent leaving the household, accident, and family sickness.

**Table 1:** Basic socio-demographics

	Wave 1 (*n* = 400)	Wave 2 (*n* = 301)	*t*-statistic	*p*-value
Female	53.8%	57%	− 0.98	0.33
Age of child (in years)	18	21	− 57.18	0.00***
Married	4.8%	17%	− 4.95	0.00***
Any children	13.0%	19.9%	− 2.12	0.03*
Deceased parent	8.3%	13%	− 2.04	0.04*
Attained primary	97.3%	99%	− 1.75	0.08
Attained secondary	82.8%	94%	− 4.64	0.00***
Enrolled in school	68.0%	50%	4.75	0.00***
Average years of education	11.8	14.3	− 15.20	0.00***
Worked in the past 12 months	76.5%	79%	− 0.92	0.36
Currently employed	35.3%	58%	− 6.06	0.00***
Subjective relative income (0–6 score)	3.0	3.0	− 0.4	0.73
Average number of negative shocks experienced	2.3	1.3	10.36	0.00***

All variables except age, average years of education, subjective relative income, and average number of negative shocks experienced are dummy variables. *P*-value of difference between the two waves is from two-tailed *t*-test. The stars represent statistical significance as follows: *p < 0.05, **p < 0.01, ***p < 0.001

In terms of household-level characteristics, and according to data from the wealth asset index, most of the houses have access to electricity, water, and a toilet. Furthermore, over 95% of households have access to a TV and a phone; 87%, to a fridge; and 60%, to a computer in the house. Many of their parents had not completed higher than secondary levels of education. As noted above, most of the fathers were construction workers, informal sector merchants, bus or taxi drivers, or carpenters, while most mothers were housekeepers, merchants/street vendors, seamstresses, or housecleaners.

Ninety-nine adolescents were lost to follow-up, resulting in an attrition rate of 24.75%. Table 5 in the appendix tests for attrition bias across observables. On average, males and adolescents who were not enrolled full-time at wave 1 were more likely to drop out at follow-up. Educational aspirations were slightly lower among those who were lost to follow-up. We observed no differences in occupational aspirations, aspirations to migrate, or other covariates such as our personality trait or risky behavior measures. Studies of attrition rates in panels cite factors such as unhealthy lifestyles or psychological distress as common predictors (Gustavson et al. 2012), but those do not seem to be at play in this instance.

We have reasons for attrition for most of these individuals (75%). This is rare as most studies lack good data on reasons for attrition. In our case, our survey team was diligent in seeking out reasons for missing respondents and was often able to get information about why they had moved away from their friends or family. Half of the missing sample had moved to a different location (within Peru and/or abroad to countries such as Spain or the USA). The rest were either not available (12%), traveling (5%), or working (4%) at the time of the interview. Parents refused to the survey in three cases, and one adolescent had a high-risk pregnancy and could not participate. Given that a significant percent of the attrition group traveled abroad to seek better jobs, we cannot attribute attrition to low aspirations or worse performance.

In peri-urban neighborhoods such as this one in Peru, it is quite common for young adults to move elsewhere to look for better jobs or other opportunities. In our case, those respondents with slightly higher education aspirations were less likely to leave the neighborhood, likely because they were enrolled in their continuing education. More generally, attrition rates for panels vary a great deal. The Rand American Life Panel (ALP), for example, has a low rate of 15%, while across studies more generally, the range is 30–70% (Gustavson et al., 2012). As such, our attrition rate of 25% for a panel of young respondents living in a relatively unstable economic context is on the low end.

### What do adolescents aspire to do in the future?

We find remarkably high aspirations in wave 1. Overall, 41% of our sample reports wanting to achieve post-graduate education (master’s degree or PhD), 47% aiming for university, and 10% aspiring for technical education. Occupational aspirations are also high, with most respondents aspiring to high-skilled professional jobs such as a lawyer, architect, or doctor. Almost all respondents (93%) aspire to migrate, with half of those wanting to migrate to another district within the same province, and a quarter to a distant country (Figs. 3, 4, and 5 in the appendix).

The main reasons for wanting to migrate are to find better education and employment opportunities or to escape high levels of crime and delinquency. We find a positive correlation across all three types of aspirations (educational, occupational, and aspirations to migrate). When asked a follow-up question about whether they can achieve their desired level of education and occupation, 89% and 96% of the sample responds affirmatively, respectively.

**Fig. 1:**
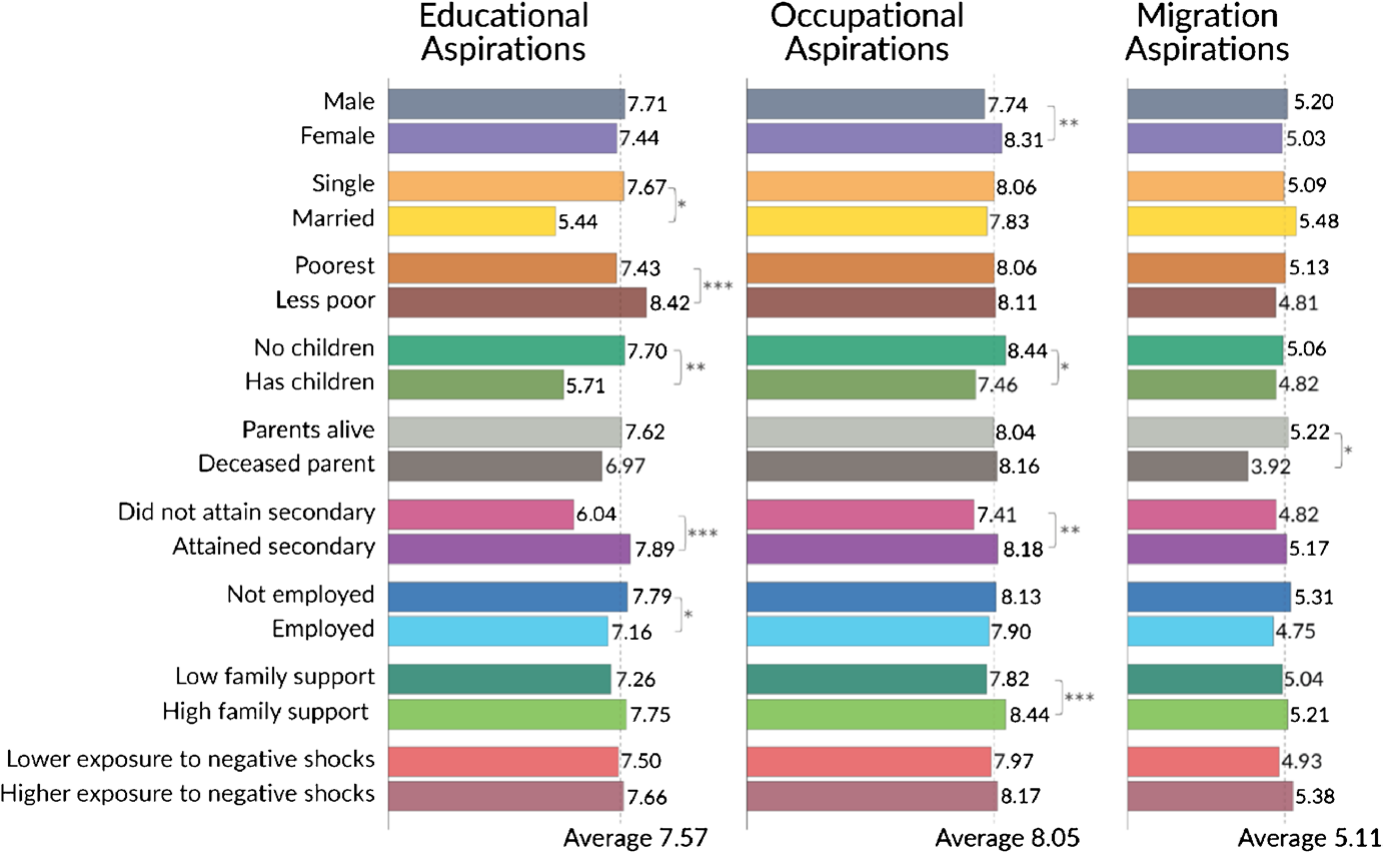
Aspirations by subgroups. Data from wave 1 was used to construct this figure. We rescaled the three types of aspirations on a 10-point scale to make it easier to compare the average level of aspirations across all three types. All individual characteristics are dummy variables. The stars represent statistical significance as follows: *p < 0.05, **p < 0.01, ***p < 0.001

Figure 1 shows how aspirations change depending on individual- and household-level characteristics. To construct this figure, we rescaled the three types of aspirations on a 10-point scale to allow for comparisons across and within types of aspirations. Several conclusions can be drawn from this figure.

First, occupational aspirations are significantly higher than educational and migration aspirations (i.e., the average level of occupational aspirations is 8.05, while it is 7.57 for the educational aspirations), and this difference is statistically significant at 0.01 level.

Second, there is some heterogeneity in aspirations depending on individual characteristics. For example, we find lower educational aspirations among adolescents who were married and those who had a child. This is not a surprise given that those who marry or have a child at such a young age have likely reduced their possibilities to continue education. We also find that educational aspirations are lower among the poor. For example, 38% of respondents from poor households aspire to achieve post-graduate, compared to 56% of respondents from highest income households which are, at most, aspiring lower middle class. Educational aspirations are also lower among those respondents who did not attain secondary education and were employed—presumably because they had to skip school or stop education all together. Occupational aspirations, meanwhile, are higher for females, those who had attained secondary education, did not have a child, and for those who lived in a household with high family support. Lastly, aspirations to migrate are lower for those adolescents who lost a parent, likely because they had to stay home to help take care of other family members.

Third, we find no difference in levels of aspirations among individuals that were highly exposed to negative shocks compared to those that did not. This is likely because negative shocks like robbery or accidents are very common in neighborhoods such as San Juan de Lurigancho, making it more likely that people adapt to them (for similar examples, see Graham, 2011). It is also at least suggestive of resilience—acquired by living in such difficult conditions—as a driving channel of the persistence of aspirations despite the obstacles.

### Do aspirations change over time?

Aspirations may change as a result of new experiences, past achievements and failures, and interactions with the social and academic environment. With time, individuals obtain a better understanding of the world and what is possible and, especially during adolescence, start to realign their behavior with the social norms of those they identify with and/or with difficult realities in their situations (Gottfredson 2002; Sebastian et al. 2008) And, as noted above, personality can evolve over time due to experience and changing preferences. At the same time, if aspirations and related goals are shaped by strong preferences and incentives, they are more likely to persist over time (Heckman and Kautz 2012).

**Fig. 2:**
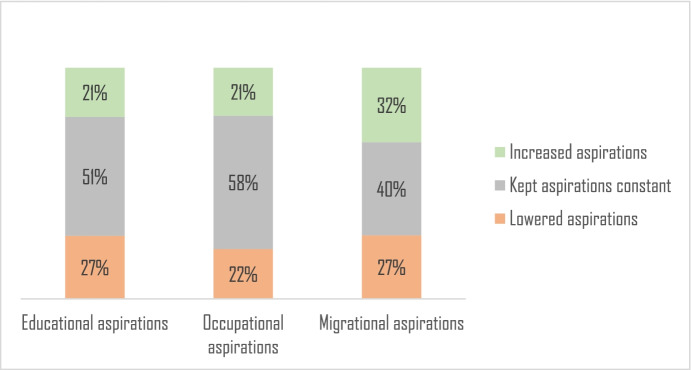
Changes in aspirations *within* individuals over time

**Fig. 3:**
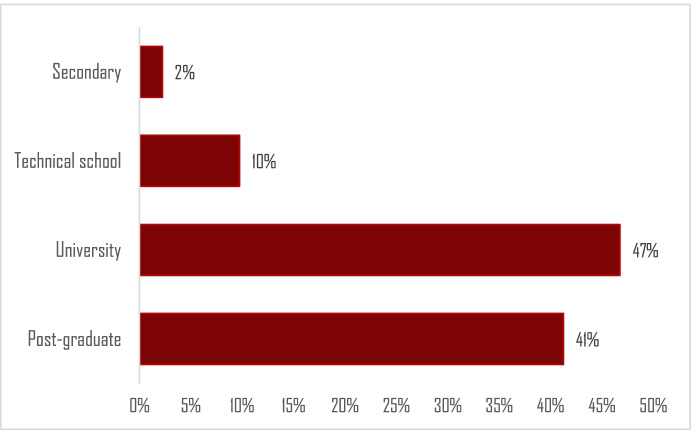
Educational aspirations in wave 1

**Fig. 4:**
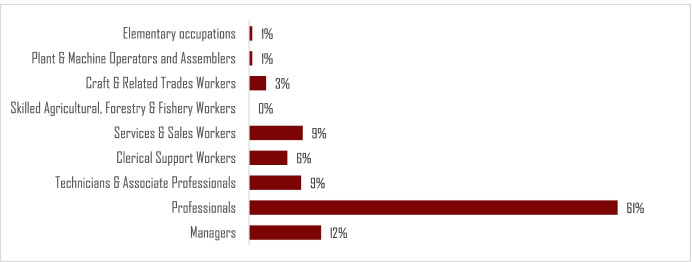
Occupational aspirations in wave 1

**Fig. 5:**
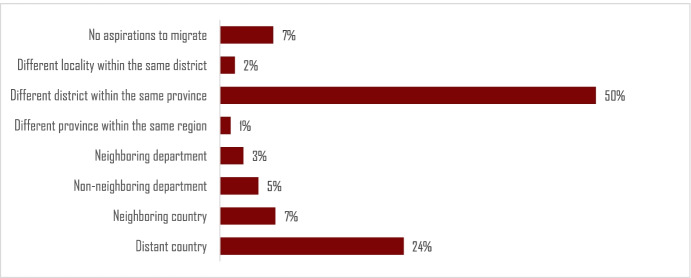
Aspirations to migrate in wave 1

In the first wave of data, our respondents were 18–19 years, point at which they likely had enough education and life experiences to internalize personal and contextual barriers to attaining these aspirations (aspirations and expectations converge). Indeed, we find that education aspirations remain relatively stable over time (Table 6 in the appendix). We calculated *t*-test for differences in average aspirations across both waves and failed to reject the null hypothesis that these two were different (*p* = 0.09). This pattern was also consistent with occupational aspirations (*p* = 0.7) and aspirations to migrate (*p* = 0.8). Half of the sample kept their aspirations constant, and the rest of the sample were evenly split between those who increased and decreased their aspirations (Fig. 2).

### Do optimists mispredict their futures?

An obvious question in this narrative is whether optimists succeed in their aspirations or mispredict their futures. Misprediction could lead to frustration and worse outcomes in the long run. Alternatively, it might simply result in no change in well-being among innately optimistic respondents, who remain so regardless of shocks or setbacks.

We are not able to measure whether or aspirations were met for most of our sample since we do not observe the complete education or labor market trajectories. Respondents are 20–22 years old at wave 2, and a third of them are still attending university. Only 4 of our respondents had completed university at the time of the second wave (all of which had aspired to attain it at wave 1). For those enrolled in university, we can only conclude that they are on track to meet their aspirations but cannot say anything about their final outcomes.

Among the individuals with lower educational aspirations who might have had time to achieve those, we observe that those individuals who aspired for technical school (*n* = 39) in wave 1 met their aspirations at wave 2. While in small sample sizes, these results suggest that the aspirations that adolescents set for themselves are realistic.

### Are aspirations and personality traits correlated?

How high an individual aspires is determined by their own beliefs about what they think they can achieve as well as their personality traits. Typically, individuals evaluate their internal and/or external constraints (one’s locus of control and/or credit) and exclude some of the unattainable options. Particularly for those living in poverty, this plays a crucial role since, very often, individual’s perceived returns are inaccurate due to imperfect information (Jensen 2010).

Mean levels of character traits in our sample are high and continue to be so in wave 2 (Table 7 in the appendix). Respondents improve in internal locus of control, self-esteem, and optimism scores over time. They are also more likely to believe that hard work will get them ahead and are more willing to take risks. On average, life satisfaction scores are relatively high in both waves. Most respondents (79%) believe that they were happier in wave 1 than they were 10 years ago, and similarly, most (76%) believed that they were happier in wave 2 than they were in wave 1. This is consistent with other work that we have done exploring optimism levels over time, where we find that respondents who are optimistic in earlier periods tend to remain optimistic in later periods and to do better in the income and education realms, even if they have suffered some negative shocks along the way (Graham and Pinto 2019; O’Connor and Graham 2019).

In Table 2, we report the pairwise correlations between all three types of aspirations and personality traits. Educational aspirations are positively correlated with self-efficacy, subjective well-being, and belief in hard work, and negatively with impatience. Occupational aspirations are negatively correlated with impatience and willingness to take risks, and positively with belief in hard work. Aspirations to migrate are correlated with belief in hard work.

**Table 2:** Correlation across types of aspirations and personality traits

	Educational aspirations	Occupational aspirations	Aspirations to migrate
Emotional symptoms (0–10 score)	− 0.04	0.04	0.07
Internal locus of control (0–6 score)	0.05	0.01	− 0.02
External locus of control (0–6 score)	− 0.06	0.01	0.06
Self-efficacy (0–15 score)	0.09*	0.04	0.05
Subjective well-being (0–8 score)	0.13***	0.01	0.03
Impatience	− 0.11**	− 0.09*	− 0.04
Belief in hard work	0.12**	0.11**	0.08*
Willingness to take risks	− 0.02	− 0.08*	0.00
Sociability	0.01	0.04	− 0.04
Self-esteem	0.04	0.04	0.04
Optimism	0.02	0.05	0.07

The first five variables are scores, with the range shown in parentheses. The remaining traits are dummy variables. Pairwise correlations were calculated pooling data from both waves. The stars represent statistical significance as follows: *p < 0.05, **p < 0.01, ***p < 0.001

### Do high aspirations lead to better human capital outcomes?

To explore this question, we look at the correlation between aspirations and future outcomes including education outcomes, time use, and adolescent’s engagement in risky behaviors such as substance use and delinquency. As shown in Table 8 in the appendix, most respondents experimented with alcohol, half of the sample smoked, and a third engaged in risky sex. Engagement in these behaviors increased over time/with age.

Results from model 1 (lagged model) are presented in Table 3. To allow for comparisons, we report standardized coefficients and only show the parameter of interest for all three types of aspirations. Educational aspirations at wave 1 predict better outcomes at wave 2. Keeping other factors constant, a 1 standard deviation increase in educational aspirations at wave 1 increases average years of education and enrollment status by 0.25 and 0.27 standard deviations. Similarly, it increases the share of time spent on school-related activities and professional development by 0.30 and 0.14 standard deviations. In contrast, a 1 standard deviation increase in educational aspirations at wave 1 decreases the likelihood of smoking and engaging in unsafe sex by 0.14 and 0.19 standard deviations. Occupational aspirations at wave 1 also predict better educational outcomes, and more time spent on school-related activities (effect sizes range from 0.12 to 0.16 standard deviations). Lastly, high aspirations to migrate are associated with more time allocated to professional development training and being less likely to carry a weapon.

**Table 3:** Model 1—lagged model: aspirations at wave 1 on outcomes at wave 2

	Educational aspirations	Occupational aspirations	Migration aspirations
	Standardized β coefficients	Standardized β coefficients	Standardized β coefficients
Average years of education	0.25** (0.09)	0.12* (0.05)	0.04 (0.06)
Enrolled full-time	0.27*** (0.06)	0.12* (0.06)	0.03 (0.06)
Share of time spent on school-related activities	0.30*** (0.06)	0.16** (0.05)	0.02 (0.06)
Pursue any professional development activities	0.14* (0.07)	0.01 (0.06)	0.13* (0.06)
Smokes cigarettes	− 0.14* (0.06)	− 0.04 (0.05)	− 0.05 (0.06)
Drinks alcohol	− 0.03 (0.05)	− 0.10 (0.06)	− 0.07 (0.05)
Risky sex	− 0.19** (0.07)	− 0.11 (0.07)	− 0.08 (0.07)
Carries weapon	0.03 (0.04)	0.02 (0.02)	− 0.10* (0.05)

Each row was a separate regression. We applied robust standard errors (in parentheses) and standardized the coefficients using the whole sample’s standard deviation. Each regression controlled for the following individual- and household-level characteristics: sex, total shocks experienced, and a household asset index constructed from information on ownership of a range of durable goods, housing characteristics, sanitation, and access to services. We also controlled for the following personality traits: emotional symptoms, locus of control (internal and external), self-efficacy, subjective well-being, impatience, and belief in hard work. The stars represent statistical significance as follows: *p < 0.05, **p < 0.01, ***p < 0.001

To obtain a better identification of the relationship between aspirations and human capital outcomes, we specify an individual fixed effect model. The results are presented in Table 4. In general, we find similar conclusions to those found in model 1, particularly for educational aspirations. Specifically, an increase in educational aspirations by one standard deviation predicts an increase in enrollment status and time spent on school activities by 0.26 and 0.21 standard deviations, respectively. These effect sizes are similar to those in model 1. High educational aspirations are also predictive of avoiding carrying weapons, which was not a significant finding in the first model. Furthermore, a one standard deviation increase in occupational aspirations reduces the likelihood of smoking by 0.18 standard deviation. The rest of the associations are not statistically significant.

**Table 4:** Model 2—individual fixed effects model

	Educational aspirations	Occupational aspirations	Migration aspirations
	Standardized β coefficients	Standardized β coefficients	Standardized β coefficients
Average years of education	0.08 (0.06)	0.01 (0.05)	0.02 (0.06)
Enrolled full-time	0.26*** (0.06)	0.08 (0.07)	0.05 (0.06)
Share of time spent on school-related activities	0.21*** (0.05)	0.03 (0.05)	0.02 (0.06)
Pursue any professional development activities	− 0.07 (0.06)	− 0.04 (0.06)	− 0.04 (0.07)
Smokes cigarettes	− 0.05 (0.06)	− 0.18** (0.06)	0.11 (0.06)
Drinks alcohol	0.07 (0.06)	− 0.07 (0.07)	0.10 (0.07)
Risky sex	− 0.00 (0.07)	− 0.02 (0.08)	0.10 (0.07)
Carries weapon	− 0.14* (0.07)	− 0.13 (0.07)	0.03 (0.06)

Each row was a separate regression. We applied robust standard errors (in parentheses) and standardized the coefficients using the whole sample’s standard deviation. Each regression controlled for the following individual- and household-level characteristics: marital status, employment status, and total shocks experienced. We also controlled for the following personality traits: emotional symptoms, locus of control (internal and external), self-efficacy, subjective well-being, impatience, and belief in hard work. The stars represent statistical significance as follows: *p < 0.05, **p < 0.01, ***p < 0.001

More generally, though, the fixed effect estimates show that the relationship between aspirations and human capital outcomes is robust to holding within person traits constant. One reason for this, noted above, is that aspirations and other traits are endogenous to the goals and preferences that frame these traits and help explain their persistence. While intuitive, this also complicates the task of identifying clear channels of causality.

**Fig. 6:**
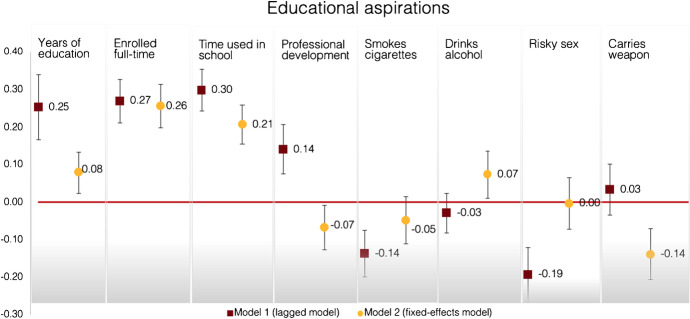
Comparing coefficients between model 1 (lagged model) and model 2 (fixed effects model). We standardized the coefficients using the whole sample’s standard deviation. Model 1 controlled for sex, total shocks experienced, household asset index, and the following personality traits: emotional symptoms, locus of control (internal and external), self-efficacy, subjective well-being, impatience, and belief in hard work. Model 2 controlled for marital status, employment status, total shocks experienced, and the same personality traits as model 1. The dot and square show the coefficient, and the bars the robust standard errors

The full specifications for model 1 and model 2 can be found in Tables 9, 10, 11, 12, 13, and 14 in the appendix, and Fig. 6  compares the coefficients from the lagged model (model 1) and the correlations with fixed effects (model 2) for educational aspirations.

## Discussion

Our research attempted to shed light on the role of aspirations in generating better future outcomes. We conducted a panel study with adolescents (18–19 years at wave 1) in a poor and near poor peri-urban neighborhood in Lima, Peru. We asked about aspirations in three domains: education, occupation, and migration, with a particular focus on education. We designed the specific measures therein based on our interest in exploring the role of hope—an understudied but important trait in our view—combined with agency as core features of aspirations and the outcomes they lead to. As such, our survey includes several questions tailored for adolescents at a point in their lives that they are making critical decisions about their futures. To our knowledge, our survey is one of a very few of its kind, with the benefits and risks that come with such exploratory data.

Our main finding was remarkably high levels of aspirations among our survey population, with over 80% of our respondents aspiring to complete university or post graduate education. Furthermore, aspirations are sticky over time, with half the sample keeping their aspirations constant 2 years later (a quarter increased them). Lastly, high aspirations are associated with better future outcomes. Respondents with high aspirations in wave 1 were more likely to have better educational- and health-related outcomes as measured by school enrollment, time allocated to school activities and professional development, and lower engagement in risky behaviors such as substance use and risky sex in wave 2. This supports our (and others’) priors that individuals with high aspirations and/or hope for the future are more likely to invest in those futures as well as to avoid behaviors that are likely to jeopardize their futures.

Our study has some limitations. First, we look at the association between aspirations and human capital outcomes using observational evidence, and thus, this paper does not claim any clear causal relation. To minimize potential endogeneity concerns, we controlled for a range of important confounders and specified a lagged model and a fixed effect model (which eliminates one major source of confounding by controlling for any unobserved time-invariant heterogeneity that may be correlated with the explanatory variables). Second, we relied on self-report measures to measure our outcomes, which could be affected by recall or reporting bias. Nevertheless, all sensitive questions (e.g., risky behaviors) were asked using a self-administered questionnaire which has been shown to reduce measurement error (Okamoto et al. 2002). Third, we are not able to measure whether or not aspirations were met for most of our sample since we do not observe the complete education or labor market trajectories. While we cannot say anything about their final human capital outcomes, our results suggest that most of our respondents are on track to meet their aspirations. Lastly, we do not have data on the respondents’ peers and their aspirations. This is particularly important during adolescence, as it is during this time when adolescents start spending more time with peers and place more value on what their peers think (and aspire to) than what families do (Blakemore and Mills 2014). We also cannot say anything about parental aspirations (aspirations that the parents have for their own children). However, anecdotal data based on interviews with those who work in this neighborhood and in Lima more generally suggest that there is a very strong shared belief in the importance of education among these parents—even though they do not have tertiary education—which in turn provides a support system for the young adults in our sample; indeed, 88% of our respondents report that their education is paid for by their parents.

While associational, our results suggest that aspirations may be an important lever to improve overall well-being and long-run outcomes. Recent evidence shows that it is possible to intervene to alter aspirations and that this, in turn, may have a causal influence on a range of human capital outcomes. For example, a study conducted in the Dominican Republic estimated that providing information on the returns to education (thus changing the perceived returns) increased completion of secondary education by 0.20–0.35 additional years (Jensen 2010). Using census data for Brazil, another study found that exposure to soap operas with strong female role models has a significant effect in lowering birth rates, with the strongest effect among women from lower socioeconomic status (La Ferrara et al. 2012). Beaman et al. (2012) show that female leadership influences adolescent girls’ career aspirations and educational attainment (Beaman et al. 2012). Lastly, a study conducted in rural Ethiopia found that playing a documentary featuring role models led to higher aspirations and better saving and investment decisions among adults (Bernard et al. 2014).

The driving channel in all these cases—as well as in other experiments—seems to be the provision of a hope channel where one previously did not exist. A recent review showed that the provision of hope in very poor populations in Africa—via the gift of a cow or some other form of livestock—improves household outcomes the following year, with hope being the most important driver of those (Haushofer and Fehr 2014). While these studies cannot reveal how long the behavioral changes last, they are, at the least, suggestive of a virtuous circle.

In Peru, education levels are high enough to drive awareness of the increasing returns to higher versus secondary education, which is likely an important incentive factor. Yet our data also suggest that traits such as optimism, self-efficacy, and internal locus of control matter independently of that. Our results show that aspirations are persistent *within* respondents, with high aspirations remaining at the same levels over the 2-year period for most respondents. While 2 years is not a long time frame, it is typically a time of many changes for individuals in their late teens and early twenties and it is striking that the aspirations of most were persistent throughout. This certainly suggests that aspirations are not just fleeting traits, as does the literature which shows that strong preference and incentive shaping goals help explain the persistence of traits like aspirations over time. The strong emphasis on educational achievement in Peru, meanwhile, likely plays a role in the persistence of aspirations in our sample of young adults. And, as noted above, we see aspirations as aimed at specific goals, but also at the achievement of broader and less well-defined ones, such as seeking a better life. The specific goals are often more tangible and achievable means to achieve the higher ones, such as achieving a certain level of education which in turn leads to a broader opportunity set.

There is much more we need to know, both about the drivers of aspirations and how the in-person and environmental factors interact in determining them, and about the consistency and durations of the channel from aspirations to better human capital outcomes. We are in the process of fielding similar versions of our surveys in different cultural and population contexts (again with low-income youth) and, if possible, in the next year or two in the same cohort in Peru. We also hope to explore the possibility of implementing scalable and cost-effective aspiration interventions in some of them.

At this juncture, though, our findings suggest that hope and aspirations matter to actual outcomes and that they may be particularly important in the context of deprived populations. This is because they do not have the same level of financial support and other advantages as wealthier ones that facilitate making key investments in their own human capital.

## Appendix

### Statistical analysis

Model 1

In the first model, we specify a lagged model as follows:

$${Outcomes}_{i,wave2}={\beta }_{0}+{\beta }_{1}{Aspirations}_{i,wave1}+{X}_{i,wave1}\Gamma +{\varepsilon }_{i,wave2}$$

where outcomes_*i*,wave2_ include the following outcomes measured at wave 2: school attainment, enrollment status, share of time dedicated to school-related activities, professional development training, or risky behaviors. Aspirations_*i*,wave1_ include each of the three types of aspirations (education, occupation, or migration), and ***X***_*i*,wave1_ is a vector of time-varying controls, including individual- and household-level characteristics, as well as personality traits. Finally, *ɛ*_*i, wave2*_ is an unobserved error term. This set-up allows us to carry out simple tests to measure the correlation between aspirations and future outcomes. If there is no correlation, we would expect *β*_1_ to be insignificant.

Model 2

We specify the second model as follows:

$${\Delta Outcomes}_{i,w2-w1}={\beta }_{1}\Delta {Aspirations}_{i,w2-w1}+\Delta {X}_{i,w2-w1}\Gamma +{\Delta \varepsilon }_{i,w2-w1}$$

Thus, we explore how changes in aspirations *within* individuals are, in turn, associated with changes in human capital outcomes between wave 1 and wave 2. Like model 1, our parameter of interest is *β*_1._

6.2 Tables and Figures Table 5

**Table 5:** Testing non-random attrition across observables

	Lost to follow up	Followed up	*t*-statistic	*p*-value
*Individual characteristics*				
Female	42%	57%	− 2.6	0.01**
Age of child (in years)	18.44	18.45	− 0.1	0.90
Married	5%	5%	0.2	0.87
Any children	12%	13%	− 0.2	0.81
Deceased parent	5%	9%	− 1.5	0.13
Worked in the past 12 months	79%	76%	0.6	0.53
Currently employed	42%	33%	1.7	0.10
Subjective relative income (0–6 score)	2.90	2.98	− 1.2	0.22
*Aspirations*				
Educational (0–10 score)	7.00	7.75	− 2.5	0.01*
Occupational (0–10 score)	7.97	8.07	− 0.5	0.62
Migration (0–10 score)	5.34	5.03	0.7	0.46
*Personality traits*				
Emotional symptoms (0–10 score)	3.67	4.16	− 1.7	0.10
Internal locus of control (0–6 score)	3.21	3.24	− 0.3	0.75
External locus of control (0–6 score)	2.53	2.49	0.3	0.75
Self-efficacy (0–15 score)	10.27	9.96	1.5	0.14
Subjective well-being (0–8 score)	4.70	4.69	0.0	0.97
*Outcomes*				
Average years of education	11.7	11.8	− 0.6	0.55
Enrolled full-time	60%	71%	− 2.0	0.05*
Share of time spent on school-related activities	34%	40%	− 2.0	0.05*
Pursue any professional development activities	33%	43%	− 1.7	0.09
Smokes cigarettes	47%	41%	1.1	0.29
Drinks alcohol	64%	68%	− 0.7	0.50
Risky sex	21%	17%	0.8	0.45
Carries weapon	2%	3%	− 0.6	0.56

We rescaled the three types of aspirations on a 10-point scale to make it easier to compare the average level of aspirations across all three types. All individual characteristics except age and subjective relative income are dummy variables. All outcomes except average years of education and share of time spent on school-related activities are dummy variables. The remaining variables are scores, with the range shown in brackets. *P*-value is from two-tailed *t*-test. The stars represent statistical significance as follows: **p* < 0.05, ***p* < 0.01, ****p* < 0.001

Table 6

**Table 6:** Changes in average aspirations over time

Aspirations	Wave 1	Wave 2	*p*-value
Education	7.8 (SD 2.3)	7.5 (SD 2.6)	0.09
Occupation	8.1 (SD 1.7)	8.1 (SD 1.9)	0.74
Migration	5.0 (SD 3.3)	4.9 (SD 3.4)	0.81

We rescaled the three types of aspirations on a 10-point scale to make it easier to compare across all three types of aspirations. *P*-value is from two-tailed *t*-test. The stars represent statistical significance as follows: * *p* < 0.05, ***p* < 0.01, ****p* < 0.001

Table 7

**Table 7:** Personality traits over time

	Wave 1 (*n* = 400)	Wave 2 (*n* = 301)	*T*-test *p*-value
Emotional symptoms (0–10 score)	4.0	4.2	0.36
Internal locus of control (0–6 score)	3.2	4.6	0.00***
External locus of control (0–6 score)	2.5	2.5	0.65
Self-efficacy (0–15 score)	10.0	10.2	0.12
Subjective well-being (0–8 score)	4.7	4.8	0.22
Impatience	0.4	0.5	0.32
Belief in hard work	3.6	3.3	0.00***
Willingness to take risks	2.6	3.0	0.00***
Sociability	3.0	3.0	0.99
Self-esteem	3.1	3.3	0.00***
Optimism	3.4	3.6	0.00***

All variables in the last six rows are dummy variables. *P*-value is from two-tailed *t*-test. The stars represent statistical significance as follows: **p* < 0.05, ***p* < 0.01, ****p* < 0.001, **p* < 0.05, ***p* < 0.01, ****p* < 0.001

Table 8

**Table 8:** Human capital outcomes over time

	Wave 1 (*n* = 400)	Wave 2 (*n* = 301)	*t*-statistic	*p*-value
Average years of education	11.8	14.3	− 15.2	0.00***
Enrolled full-time	68%	50%	4.8	0.00***
Share of time spent on school-related activities	39%	28%	4.6	0.00***
Pursue any professional development activities	41%	55%	− 4.0	0.20
Smokes cigarettes	42%	49%	− 1.7	0.09
Drinks alcohol	67%	81%	− 4.3	0.00***
Risky sex	18%	31%	− 3.8	0.00***
Carries weapon	3%	2%	0.4	0.72

Table 9

**Table 9:** Model 1 (lagged model). Educational aspirations

Variables	Average years of education	Enrolled full-time	Share of time spent on school-related activities	Pursue any professional development activities	Smokes cigarettes	Drinks alcohol	Risky sex	Carries weapon
Educational aspirations at wave 1	0.25**	0.27***	0.30***	0.14*	− 0.14*	− 0.03	− 0.19**	0.03
	(0.09)	(0.06)	(0.06)	(0.07)	(0.06)	(0.05)	(0.07)	(0.04)
Female	0.01	− 0.11	− 0.18**	− 0.06	− 0.30***	− 0.12*	− 0.11	− 0.08
	(0.06)	(0.06)	(0.06)	(0.06)	(0.06)	(0.05)	(0.07)	(0.05)
Household asset index	0.16**	0.25***	0.25***	0.03	0.02	0.01	− 0.16*	− 0.05
	(0.06)	(0.05)	(0.05)	(0.06)	(0.06)	(0.04)	(0.07)	(0.07)
Total shocks experienced	0.02	− 0.05	− 0.10	0.05	0.01	0.04	0.01	− 0.03
	(0.06)	(0.06)	(0.06)	(0.06)	(0.06)	(0.04)	(0.07)	(0.03)
Emotional symptoms	0.22**	0.22***	0.20***	0.09	0.07	0.06	0.06	0.05
	(0.07)	(0.06)	(0.06)	(0.06)	(0.06)	(0.05)	(0.07)	(0.06)
Internal locus of control	0.01	− 0.04	− 0.03	− 0.03	0.18*	0.05	0.15	0.14
	(0.07)	(0.08)	(0.09)	(0.09)	(0.09)	(0.07)	(0.09)	(0.08)
External locus of control	− 0.03	− 0.03	− 0.02	− 0.04	− 0.08	0.00	− 0.09	− 0.19*
	(0.06)	(0.07)	(0.06)	(0.07)	(0.06)	(0.05)	(0.07)	(0.09)
Self-efficacy	0.05	0.04	0.04	− 0.03	0.02	0.04	− 0.02	0.01
	(0.05)	(0.06)	(0.06)	(0.06)	(0.07)	(0.04)	(0.07)	(0.07)
Subjective well-being	0.04	0.02	0.03	− 0.05	− 0.03	0.05	− 0.04	− 0.02
	(0.06)	(0.05)	(0.05)	(0.06)	(0.06)	(0.05)	(0.07)	(0.05)
Impatience	− 0.06	− 0.06	− 0.03	0.05	− 0.04	− 0.10*	− 0.07	− 0.08
	(0.06)	(0.06)	(0.06)	(0.06)	(0.06)	(0.04)	(0.07)	(0.05)
Belief in hard work	− 0.01	0.02	− 0.00	0.05	0.01	− 0.01	0.11	0.07
	(0.05)	(0.07)	(0.06)	(0.06)	(0.05)	(0.07)	(0.07)	(0.05)
Constant	0.57***	− 0.24**	− 0.20**	0.11	0.18*	− 0.10	0.23**	0.05
	(0.07)	(0.07)	(0.07)	(0.08)	(0.09)	(0.06)	(0.09)	(0.09)
Observations	248	300	301	301	297	296	291	296
*R*-squared	0.16	0.20	0.23	0.04	0.11	0.06	0.07	0.05

We applied robust standard errors (in paretheses) and standardized the coefficients using the whole sample’s standard deviation. The stars represent statistical significance as follows: **p* < 0.05, ***p* < 0.01, ****p* < 0.001

Table 10

**Table 10:** Model 1 (lagged model). Occupational aspirations

Variables	Average years of education	Enrolled full-time	Share of time spent on school-related activities	Pursue any professional development activities	Smokes cigarettes	Drinks alcohol	Risky sex	Carries weapon
Occupational aspirations at wave 1	0.12*	0.12*	0.16**	0.01	− 0.04	− 0.10	− 0.11	0.02
	(0.05)	(0.06)	(0.05)	(0.06)	(0.05)	(0.06)	(0.07)	(0.02)
Female	− 0.02	− 0.14*	− 0.22***	− 0.07	− 0.29***	− 0.10*	− 0.09	− 0.09
	(0.06)	(0.06)	(0.06)	(0.06)	(0.06)	(0.05)	(0.07)	(0.05)
Household asset index	0.20**	0.30***	0.31***	0.05	− 0.01	− 0.01	− 0.20**	− 0.05
	(0.06)	(0.05)	(0.05)	(0.06)	(0.06)	(0.04)	(0.07)	(0.07)
Total shocks experienced	0.05	− 0.04	− 0.09	0.05	− 0.01	0.03	0.00	− 0.03
	(0.06)	(0.06)	(0.06)	(0.06)	(0.06)	(0.04)	(0.07)	(0.03)
Emotional symptoms	0.21**	0.21***	0.19**	0.09	0.08	0.06	0.06	0.05
	(0.07)	(0.06)	(0.06)	(0.06)	(0.06)	(0.05)	(0.07)	(0.06)
Internal locus of control	0.01	− 0.05	− 0.05	− 0.03	0.20*	0.07	0.16	0.14
	(0.07)	(0.09)	(0.09)	(0.09)	(0.09)	(0.07)	(0.09)	(0.08)
External locus of control	− 0.03	− 0.03	− 0.02	− 0.05	− 0.08	− 0.00	− 0.09	− 0.19*
	(0.06)	(0.07)	(0.07)	(0.07)	(0.07)	(0.05)	(0.07)	(0.09)
Self-efficacy	0.07	0.06	0.07	− 0.03	0.00	0.04	− 0.03	0.01
	(0.05)	(0.06)	(0.06)	(0.06)	(0.07)	(0.04)	(0.07)	(0.07)
Subjective well-being	0.05	0.04	0.05	− 0.04	− 0.04	0.04	− 0.04	− 0.02
	(0.07)	(0.06)	(0.05)	(0.06)	(0.06)	(0.05)	(0.07)	(0.05)
Impatience	− 0.07	− 0.07	− 0.04	0.04	− 0.02	− 0.11**	− 0.05	− 0.08
	(0.06)	(0.06)	(0.06)	(0.06)	(0.06)	(0.04)	(0.07)	(0.05)
Belief in hard work	− 0.02	0.02	0.00	0.06	0.00	0.00	0.12	0.07
	(0.06)	(0.07)	(0.06)	(0.07)	(0.05)	(0.07)	(0.07)	(0.05)
Constant	0.62***	− 0.22**	− 0.18*	0.12	0.18*	− 0.10	0.22*	0.05
	(0.07)	(0.08)	(0.08)	(0.08)	(0.09)	(0.06)	(0.09)	(0.09)
Observations	245	297	298	298	295	294	289	294
*R*-squared	0.13	0.16	0.19	0.02	0.09	0.07	0.06	0.05

We applied robust standard errors (in paretheses) and standardized the coefficients using the whole sample’s standard deviation. The stars represent statistical significance as follows: **p* < 0.05, ***p* < 0.01, ****p* < 0.001

Table 11

**Table 11:** Model 1 (lagged model). Migration aspirations

Variables	Average years of education	Enrolled full-time	Share of time spent on school-related activities	Pursue any professional development activities	Smokes cigarettes	Drinks alcohol	Risky sex	Carries weapon
Migration aspirations at wave 1	0.04	0.03	0.02	0.13*	− 0.05	− 0.07	− 0.08	− 0.10*
	(0.06)	(0.06)	(0.06)	(0.1)	(0.06)	(0.05)	(0.07)	(0.05)
Female	0.02	− 0.12	− 0.19**	− 0.07	− 0.29***	− 0.13*	− 0.10	− 0.11*
	(0.07)	(0.06)	(0.07)	(0.1)	(0.07)	(0.06)	(0.07)	(0.06)
Household asset index	0.21**	0.30***	0.31***	0.07	0.00	− 0.02	− 0.22**	− 0.07
	(0.07)	(0.05)	(0.05)	(0.1)	(0.06)	(0.05)	(0.07)	(0.07)
Total shocks experienced	0.06	0.00	− 0.03	0.07	− 0.00	0.04	− 0.04	− 0.05
	(0.07)	(0.06)	(0.06)	(0.1)	(0.07)	(0.05)	(0.07)	(0.04)
Emotional symptoms	0.21**	0.22**	0.17**	0.04	0.06	0.06	0.11	0.00
	(0.08)	(0.07)	(0.06)	(0.1)	(0.07)	(0.06)	(0.08)	(0.05)
Internal locus of control	0.03	− 0.04	− 0.05	− 0.01	0.20*	0.06	0.24**	0.15
	(0.08)	(0.09)	(0.09)	(0.1)	(0.10)	(0.07)	(0.09)	(0.08)
External locus of control	0.01	− 0.06	− 0.05	− 0.07	− 0.10	− 0.02	− 0.11	− 0.16
	(0.06)	(0.07)	(0.07)	(0.1)	(0.07)	(0.05)	(0.08)	(0.08)
Self-efficacy	0.04	0.07	0.06	− 0.07	0.02	0.05	− 0.03	0.05
	(0.05)	(0.06)	(0.06)	(0.1)	(0.07)	(0.04)	(0.07)	(0.07)
Subjective well-being	0.05	0.05	0.07	− 0.02	− 0.01	0.05	− 0.05	− 0.08
	(0.07)	(0.06)	(0.06)	(0.1)	(0.06)	(0.05)	(0.08)	(0.04)
Impatience	− 0.08	− 0.07	− 0.05	− 0.00	− 0.04	− 0.13**	− 0.08	− 0.12*
	(0.06)	(0.06)	(0.06)	(0.1)	(0.06)	(0.04)	(0.07)	(0.05)
Belief in hard work	− 0.01	0.02	0.00	0.10	0.00	0.01	0.10	0.05
	(0.06)	(0.07)	(0.06)	(0.1)	(0.06)	(0.08)	(0.07)	(0.04)
Constant	0.64***	− 0.21*	− 0.18*	0.10	0.22*	− 0.09	0.33***	0.05
	(0.08)	(0.08)	(0.08)	(0.1)	(0.10)	(0.07)	(0.10)	(0.10)
Observations	217	265	266	266	263	262	258	262
*R*-squared	0.12	0.16	0.17	0.04	0.10	0.08	0.08	0.09

We applied robust standard errors (in paretheses) and standardized the coefficients using the whole sample’s standard deviation. The stars represent statistical significance as follows: **p* < 0.05, ***p* < 0.01, ****p* < 0.001

Table 12

**Table 12:** Model 2 (individual fixed effects model). Educational aspirations

Variables	Average years of education	Enrolled full-time	Share of time spent on school-related activities	Pursue any professional development activities	Smokes cigarettes	Drinks alcohol	Risky sex	Carries weapon
Educational aspirations	0.08	0.26***	0.21***	− 0.07	− 0.05	0.07	− 0.00	− 0.14*
	(0.06)	(0.06)	(0.05)	(0.06)	(0.06)	(0.06)	(0.07)	(0.07)
Marital status	0.02	− 0.17***	− 0.14***	− 0.04	− 0.05	− 0.08	0.15*	− 0.13
	(0.05)	(0.04)	(0.04)	(0.06)	(0.05)	(0.04)	(0.06)	(0.07)
Employment	0.05	− 0.12*	− 0.27***	0.16**	0.03	0.00	0.01	− 0.10
	(0.05)	(0.05)	(0.04)	(0.06)	(0.04)	(0.05)	(0.06)	(0.06)
Total shocks experienced	− 0.20***	0.10	0.03	0.03	− 0.05	0.05	− 0.03	0.02
	(0.06)	(0.06)	(0.05)	(0.07)	(0.05)	(0.06)	(0.06)	(0.04)
Emotional symptoms	− 0.05	− 0.03	− 0.06	0.03	− 0.05	− 0.01	0.11	0.10
	(0.07)	(0.07)	(0.06)	(0.08)	(0.07)	(0.07)	(0.09)	(0.08)
Internal locus of control	0.32***	− 0.07	− 0.05	0.04	0.06	0.02	0.11*	− 0.02
	(0.04)	(0.04)	(0.04)	(0.05)	(0.04)	(0.05)	(0.05)	(0.04)
External locus of control	0.01	0.01	0.02	0.05	0.07	0.02	− 0.00	0.13
	(0.05)	(0.06)	(0.05)	(0.06)	(0.04)	(0.05)	(0.05)	(0.08)
Self-efficacy	0.02	− 0.09	− 0.11*	0.13*	− 0.05	− 0.04	0.01	0.01
	(0.05)	(0.05)	(0.05)	(0.06)	(0.05)	(0.06)	(0.07)	(0.07)
Subjective well-being	0.01	0.12*	0.08	0.14*	− 0.03	− 0.07	− 0.01	0.10
	(0.06)	(0.06)	(0.05)	(0.06)	(0.05)	(0.06)	(0.06)	(0.06)
Impatience	− 0.03	0.07	0.10*	− 0.08	0.05	0.07	0.03	0.06
	(0.05)	(0.05)	(0.04)	(0.05)	(0.05)	(0.05)	(0.05)	(0.07)
Belief in hard work	− 0.20***	0.03	0.07	− 0.12*	− 0.01	0.15**	− 0.06	− 0.02
	(0.05)	(0.06)	(0.05)	(0.06)	(0.04)	(0.06)	(0.06)	(0.05)
Constant	0.02***	− 0.00	0.00***	− 0.00***	0.00	0.00	− 0.00	− 0.00
	(0.00)	(0.00)	(0.00)	(0.00)	(0.00)	(0.00)	(0.00)	(0.00)
Observations	645	700	701	701	688	676	678	688
*R*-squared	0.43	0.20	0.26	0.10	0.04	0.06	0.10	0.07
Participants	397	400	400	400	398	395	395	399

We applied robust standard errors (in paretheses) and standardized the coefficients using the whole sample’s standard deviation. The stars represent statistical significance as follows: **p* < 0.05, ***p* < 0.01, ****p* < 0.001

Table 13

**Table 13:** Model 2 (individual fixed effects model). Occupational aspirations

Variables	Average years of education	Enrolled full-time	Share of time spent on school-related activities	Pursue any professional development activities	Smokes cigarettes	Drinks alcohol	Risky sex	Carries weapon
Occupational aspirations	0.01	0.08	0.03	− 0.04	− 0.18**	− 0.07	− 0.02	− 0.13
	(0.05)	(0.07)	(0.05)	(0.06)	(0.06)	(0.07)	(0.08)	(0.07)
Marital status	0.02	− 0.15**	− 0.12*	− 0.05	− 0.04	− 0.05	0.16*	− 0.17
	(0.06)	(0.06)	(0.05)	(0.07)	(0.06)	(0.05)	(0.07)	(0.10)
Employment	0.04	− 0.08	− 0.24***	0.16*	0.02	− 0.02	0.04	− 0.08
	(0.06)	(0.06)	(0.05)	(0.07)	(0.04)	(0.06)	(0.06)	(0.07)
Total shocks experienced	− 0.22***	0.05	− 0.00	0.04	− 0.04	0.08	− 0.08	0.03
	(0.07)	(0.07)	(0.06)	(0.08)	(0.05)	(0.06)	(0.07)	(0.04)
Emotional symptoms	− 0.13	− 0.04	− 0.09	− 0.05	− 0.01	− 0.03	0.16	0.07
	(0.07)	(0.08)	(0.07)	(0.10)	(0.07)	(0.07)	(0.11)	(0.06)
Internal locus of control	0.32***	-0.11*	− 0.09*	0.01	0.05	0.02	0.07	− 0.00
	(0.04)	(0.05)	(0.04)	(0.06)	(0.04)	(0.05)	(0.06)	(0.05)
External locus of control	− 0.04	− 0.00	− 0.00	0.09	0.05	− 0.02	0.03	0.13
	(0.05)	(0.07)	(0.06)	(0.06)	(0.04)	(0.05)	(0.06)	(0.09)
Self-efficacy	− 0.04	− 0.12	− 0.14**	0.13	− 0.00	− 0.04	0.00	− 0.02
	(0.06)	(0.07)	(0.05)	(0.07)	(0.05)	(0.06)	(0.07)	(0.09)
Subjective well-being	0.00	0.13*	0.09	0.15*	− 0.06	− 0.09	0.03	0.07
	(0.06)	(0.06)	(0.05)	(0.07)	(0.05)	(0.06)	(0.06)	(0.06)
Impatience	− 0.05	0.07	0.06	− 0.05	0.06	0.05	0.04	− 0.01
	(0.06)	(0.06)	(0.05)	(0.06)	(0.05)	(0.06)	(0.06)	(0.06)
Belief in hard work	− 0.19**	0.06	0.05	− 0.16*	− 0.02	0.17**	− 0.07	0.00
	(0.07)	(0.06)	(0.05)	(0.06)	(0.04)	(0.06)	(0.06)	(0.06)
Constant	0.02***	0.02***	0.02***	− 0.02***	− 0.01***	− 0.00	− 0.01	− 0.01*
	(0.01)	(0.00)	(0.00)	(0.00)	(0.00)	(0.00)	(0.00)	(0.00)
Observations	603	645	645	645	635	623	625	635
*R*-squared	0.44	0.15	0.21	0.10	0.09	0.09	0.11	0.08
Participants	396	399	399	399	396	390	391	397

We applied robust standard errors (in paretheses) and standardized the coefficients using the whole sample’s standard deviation. The stars represent statistical significance as follows: **p* < 0.05, ***p* < 0.01, ****p* < 0.001

Table 14

**Table 14:** Model 2 (individual fixed effects model). Migration aspirations

Variables	Average years of education	Enrolled full-time	Share of time spent on school-related activities	Pursue any professional development activities	Smokes cigarettes	Drinks alcohol	Risky sex	Carries weapon
Migration aspirations	0.02	0.05	0.02	− 0.04	0.11	0.10	0.10	0.03
	(0.06)	(0.06)	(0.06)	(0.07)	(0.06)	(0.07)	(0.07)	(0.06)
Marital status	0.01	− 0.23***	− 0.22***	0.01	− 0.06	− 0.04	0.16*	− 0.18
	(0.06)	(0.06)	(0.06)	(0.06)	(0.07)	(0.05)	(0.08)	(0.10)
Employment	0.08	− 0.12	− 0.28***	0.21**	0.02	− 0.00	0.03	− 0.12
	(0.06)	(0.07)	(0.06)	(0.07)	(0.05)	(0.06)	(0.06)	(0.07)
Total shocks experienced	− 0.17*	0.03	− 0.07	0.06	− 0.03	0.07	0.05	0.06
	(0.07)	(0.07)	(0.06)	(0.08)	(0.06)	(0.08)	(0.08)	(0.05)
Emotional symptoms	− 0.00	− 0.11	− 0.12	0.04	− 0.04	0.01	0.00	0.12
	(0.07)	(0.09)	(0.08)	(0.09)	(0.08)	(0.08)	(0.11)	(0.12)
Internal locus of control	0.29***	− 0.08	− 0.09	0.00	0.09*	0.02	0.11	0.01
	(0.05)	(0.05)	(0.05)	(0.06)	(0.04)	(0.06)	(0.06)	(0.05)
External locus of control	− 0.03	0.07	0.06	0.09	0.08	0.03	− 0.03	0.08
	(0.06)	(0.08)	(0.06)	(0.06)	(0.06)	(0.07)	(0.07)	(0.10)
Self-efficacy	0.00	− 0.01	− 0.03	0.16*	− 0.12*	− 0.03	0.00	0.04
	(0.06)	(0.07)	(0.06)	(0.07)	(0.05)	(0.07)	(0.09)	(0.08)
Subjective well-being	0.05	0.15	0.08	0.09	− 0.06	− 0.02	− 0.01	0.13
	(0.07)	(0.08)	(0.06)	(0.07)	(0.07)	(0.08)	(0.07)	(0.09)
Impatience	− 0.08	0.06	0.09	− 0.12	0.09	0.07	0.01	0.11
	(0.07)	(0.07)	(0.06)	(0.06)	(0.06)	(0.07)	(0.06)	(0.08)
Belief in hard work	− 0.19**	− 0.03	− 0.01	− 0.11	0.04	0.18**	− 0.04	0.04
	(0.06)	(0.07)	(0.05)	(0.06)	(0.05)	(0.06)	(0.07)	(0.06)
Constant	− 0.01	0.00	0.00	− 0.02***	0.04***	0.02***	0.02***	0.01
	(0.01)	(0.00)	(0.00)	(0.00)	(0.00)	(0.00)	(0.00)	(0.00)
Observations	558	596	596	596	586	575	579	587
R-squared	0.40	0.13	0.21	0.12	0.08	0.08	0.08	0.08
Participants	379	385	385	385	381	376	379	382

We applied robust standard errors (in paretheses) and standardized the coefficients using the whole sample’s standard deviation. The stars represent statistical significance as follows: **p* < 0.05, ***p* < 0.01, ****p* < 0.001

Figure 3

Figure 4

Figure 5

Figure 6

## Footnotes

1 In 2018, net enrollment rate was over 95% for primary education, and 89.3% for secondary education. Gross enrollment ratio for tertiary education in 2017 was 70.7% (UNESCO, 2018).

2 In addition to informal interviews, one of the authors also received a number of comments supporting this pattern when presenting the first-round results at the IIN in November 2018.
